# Understanding conservationists’ perspectives on the new‐conservation debate

**DOI:** 10.1111/cobi.12811

**Published:** 2016-11-30

**Authors:** George Holmes, Chris Sandbrook, Janet A. Fisher

**Affiliations:** ^1^ School of Earth and Environment University of Leeds Woodhouse Lane, Leeds LS2 9JT U.K.; ^2^ UNEP World Conservation Monitoring Centre 219 Huntingdon Road Cambridge CB3 0DL U.K.; ^3^ Department of Geography University of Cambridge Downing Place Cambridge CB2 3EN U.K.; ^4^ School of GeoSciences University of Edinburgh Drummond Street Edinburgh EH8 9XP U.K.

**Keywords:** human well‐being, markets, nature, neoliberal conservation, Q methodology, values, wilderness, bienestar humano, conservación neoliberal, mercados, metodología Q, naturaleza, valores, vida silvestre

## Abstract

A vibrant debate about the future direction of biodiversity conservation centers on the merits of the so‐called new conservation. Proponents of the new conservation advocate a series of positions on key conservation ideas, such as the importance of human‐dominated landscapes and conservation's engagement with capitalism. These have been fiercely contested in a debate dominated by a few high‐profile individuals, and so far there has been no empirical exploration of existing perspectives on these issues among a wider community of conservationists. We used Q methodology to examine empirically perspectives on the new conservation held by attendees at the 2015 International Congress for Conservation Biology (ICCB). Although we identified a consensus on several key issues, 3 distinct positions emerged: in favor of conservation to benefit people but opposed to links with capitalism and corporations, in favor of biocentric approaches but with less emphasis on wilderness protection than prominent opponents of new conservation, and in favor of the published new conservation perspective but with less emphasis on increasing human well‐being as a goal of conservation. Our results revealed differences between the debate on the new conservation in the literature and views held within a wider, but still limited, conservation community and demonstrated the existence of at least one viewpoint (in favor of conservation to benefit people but opposed to links with capitalism and corporations) that is almost absent from the published debate. We hope the fuller understanding we present of the variety of views that exist but have not yet been heard, will improve the quality and tone of debates on the subject.

## Abstract (es)

Entendiendo las Perspectivas de los Conservacionistas sobre el Debate de la Nueva Conservación

**Resumen**

Un debate vibrante sobre la futura dirección de la conservación de la biodiversidad se centra en los méritos de la supuesta nueva conservación. Quienes proponen la nueva conservación abogan por una serie de posiciones sobre ideas clave de conservación, como la importancia de los paisajes dominados por humanos y la participación de la conservación dentro del capitalismo. Estas ideas han sido disputadas ferozmente en un debate dominado por unos cuantos individuos de alto perfil y hasta ahora no ha habido una exploración empírica de las perspectivas existentes sobre estos temas entre una comunidad más amplia de conservacionistas. Utilizamos la metodología Q para examinar empíricamente las perspectivas sobre la nueva conservación que realizaron quienes asistieron al Congreso Internacional para la Biología de la Conservación 2015 (CIBC). Aunque identificamos un consenso sobre varios temas importantes, emergieron tres posiciones distintas: a favor de la conservación para beneficiar a las personas pero en contra de los vínculos con el capitalismo y las corporaciones, a favor de las estrategias biocéntricas pero con menos énfasis en la protección de la vida silvestre que los oponentes prominentes de la nueva conservación, y a favor de la perspectiva publicada de la nueva conservación pero con menos énfasis en el creciente bienestar humano como meta de la conservación. Nuestros resultados revelaron diferencias entre el debate sobre la nueva conservación en la literatura y los puntos de vista dentro de una comunidad de la conservación más amplia pero todavía limitada y demostró la existencia de por lo menos un punto de vista (a favor de la conservación para beneficiar a las personas pero en contra de los vínculos con el capitalismo y las corporaciones) que está casi ausente del debate publicado. Esperamos que el entendimiento más completo que presentamos de la variedad de puntos de vista que existen pero aún no han sido escuchados mejorará la calidad y el todo de los debates sobre el tema.

## Introduction

“Conservation in the Anthropocene” (Kareiva et al. 2012) triggered a vibrant, and often contentious, debate about the future of biodiversity conservation. This debate, over what has become known as the new conservation, has unfolded through a series of position and opinion pieces that are mostly either in favor of the new conservation (Kareiva et al. 2012; Kareiva & Marvier 2012) or against it (Greenwald et al. 2013; Noss et al. 2013; Soulé 2013; Doak et al. 2014; Miller et al. 2014). Several pieces analyzed the nature and tone of the debate (Hunter et al. 2014; Tallis & Lubchenco 2014). Although it has extended into the broader conservation community, the debate's public manifestations have been “dominated by only a few voices, nearly all of them men's” (Tallis & Lubchenco 2014: 27), and no attempt has been made to describe views from a wider community of conservationists. This has led hundreds of signatories to back Tallis and Lubchenco's (2014) call for a new chapter in the debate based on a wider range of views.

Originally proposed in an essay for The Breakthrough Institute (Kareiva et al. 2012) and further developed in later articles (Kareiva & Marvier 2012), the new conservation is based on a series of core principles and values (described by its authors as functional and normative postulates, respectively) for conservation in the 21st century (Table 1). The new conservation postulates are an attempt to update Soulé’s (1985) foundational functional postulates for conservation. They draw on developments in the conservation sciences and react to what Kareiva and Marvier (2012) see as Soulé’s damaging inattention to human well‐being.

**Table 1:** Functional and normative postulates for the new conservation as proposed in Kareiva and Marvier

**Functional postulate**
“ ‘pristine nature,’ untouched by human influences, does not exist”
“the fate of nature and that of people are deeply intertwined”
“nature can be surprisingly resilient”
“human communities can avoid the tragedy of the commons”
“local conservation efforts are deeply connected to global forces”
**Normative postulate**
“conservation must occur within human‐altered landscapes”
“conservation will be a durable success only if people support conservation goals”
“conservationists must work with corporations”
“conservation must not infringe on human rights and must embrace the principles of fairness and gender equity”

In response, authors who might be called traditional conservationists contend, *inter alia*, that new conservation exaggerates nature's resilience, that its embrace of economic growth ignores fundamental planetary limits, and that there are many almost‐intact wildernesses worth saving, which are neglected by a greater focus on conserving human‐dominated places (Jacquet 2013; Noss et al. 2013; Soulé 2013; Doak et al. 2014; Miller et al. 2014; Wilson 2016). Traditional conservationists also argue that most conservation already takes place in human‐dominated places. In contrast to Kareiva and Marvier's (2012) assertion, Greenwald et al. (2013) argue that conservation has long held concerns for human well‐being, and this was mentioned in Soulé’s (1985) seminal article.

The antagonism is partly because the debate on new conservation is not just about how conservation should be done but also about different ethical values that underpin why conservation should be done and for whom (Hunter et al. 2014). New conservation is more anthropocentric, emphasizing the benefits of nature to humans and prioritizing the emergent properties of ecosystems that provide these, such as stability and productivity. Traditional conservation is more biocentric, emphasizing the intrinsic value of nature and prioritizing issues of species diversity and extinction. These values are often implicit rather than explicit in key position papers (Hunter et al. 2014).

Conservation has a history of plural views driving different framings of what conservation is, and what it is for (Mace 2014), and these longer‐running debates are reflected in the current new versus traditional conservation debate (Holmes 2015). There has been a long debate about whether poverty alleviation in conservation is a damaging distraction, an ethically justifiable addition to the mission of conservationists, or a vital tool to make conservation more effective (Roe 2008). Similarly, there have been disputes over whether true wilderness exists and whether it is a useful or harmful concept for conservation (Callicot & Nelson 1998). Conservationists variously advocate for and critique working with corporations and capitalism (Brockington & Duffy 2010). What is new in the new‐conservation debate is the way these and other issues have been packaged into just 2 opposing positions on why, how, and what to conserve (Holmes 2015). Meanwhile, other relevant debates in conservation social science, such as those on biocultural diversity, remain absent.

One substantial body of social science literature emerging in recent years, which is particularly relevant to many key themes in the new conservation, is that on neoliberal conservation. This explores the increasing integration between conservation and capitalism, considering the mechanisms by which such integration has taken place (e.g., payments for ecosystem services, biodiversity offsetting, and ecotourism), the claims of synergies between conservation and capitalism that underpin these mechanisms, and the role of major conservation nongovernmental organizations (NGOs) in promoting such mechanisms (Igoe & Brockington 2007; Brockington & Duffy 2010). These claimed synergies are part of the new‐conservation discourse, which warns against “scolding capitalism” (Kareiva et al. 2012) and advocates working with corporations not as a “necessary evil” but because they “can be a positive force for conservation” (Kareiva & Marvier 2012: 967). The critical literature on neoliberal conservation originates from diverse authors, including political ecologists (Igoe & Brockington 2007), conservation biologists (McCauley 2015), and mixtures of the 2 (Redford & Adams 2009). It has direct relevance to the new‐conservation debate, but explicit cross‐referencing between the two is rare (but see Spash 2015).

We sought to expand the debate about new conservation beyond the voices of a few prominent individuals by empirically examining the range of positions that exist among a wider group of conservationists, sampled from an international conservation conference. Accordingly, we aimed to evaluate the extent to which a particular group of conservationists share the views espoused in the public debate or adopt more nuanced or contrasting positions.

## Methods

### Q Methodology

We used Q methodology to undertake a systematic analysis of the perspectives of conservation professionals attending the 2015 International Congress on Conservation Biology (ICCB) in France. This method is growing in popularity for examinations of structure and form within subjective opinions and discourses, and it has been increasingly applied to conservation research in recent years (e.g., Sandbrook et al. 2011; Cairns et al. 2014; Fisher & Brown 2014). It combines the qualitative study of perceptions with the statistical rigor of quantitative techniques (McKeown & Thomas 1998; Watts & Stenner 2012) and requires respondents to arrange statements drawn from the public discourse on the research topic onto a grid to reflect their views. The method is used to identify particular subjective positions, identified as factors, and how these are shared by people. It also enables the detailed analysis and comparison of the composition of these positions. The prevalence of positions in a population, which is the domain of conventional surveys, is not of concern with Q methodology. Accordingly, Q is designed for small numbers of participants and does not require a random sample (McKeown & Thomas 1998). Watts and Stenner (2012) provide a comprehensive explanation of Q methodology.

### Q Statements

A Q study starts by defining statements. We identified potential statements from the peer‐reviewed literature that introduces, critiques, and defends ideas associated with the new conservation (Supporting Information). To identify material to review, we started with the key articles that launched the new‐conservation debate (e.g., Kareiva et al. 2012; Kareiva & Marvier 2012) and then used Google Scholar to identify all articles citing this work, discarding those that were clearly not relevant. We selected candidate Q statements from the articles covering the major themes of the new conservation literature. The Q statements must span the range of existing positions and be concise and clear, such that respondents can place them instinctively. We chose 38 statements from an initial list of 108 by eliminating redundant statements, the meaning of which was more effectively conveyed elsewhere. Some statements were rephrased for clarity or to reverse their meaning to give a balanced set of statements (called a Q set). We tested this set with 3 respondents (two academics working on conservation issues and a representative from an international conservation NGO). Minor alterations for clarity were undertaken following the pilot phase.

### Recruiting Q Participants

Our respondents were delegates at the ICCB. This congress is the main international event of the Society for Conservation Biology (http://www.conbio.org/AboutUs/). We chose attendees of this event to capture views on the new‐conservation debate from a wider group of respondents than those who had previously contributed publicly to the debate. However, our respondents were likely to have read or heard about it because they are part of the conservation mainstream, including academics and practitioners from major NGOs. The ICCB is the largest academic conservation conference in the world. The 2015 conference attracted roughly 2000 delegates from about 100 countries, making it an ideal venue for our study. One plenary session was a debate between Peter Kareiva and ecological economist Clive Spash on the new conservation, an event that likely prompted delegates to think about these issues. The attendees at the ICCB, and correspondingly the data we gathered, did not span the entire breadth that may exist within conservation on these issues. Many key voices, such as indigenous groups and rural residents of the global South, are significantly underrepresented at such events. Nevertheless, sampling the conference delegates allowed us to meet our objective of surveying views from a wider group of conservationists than those who have dominated the public debate on the new conservation.

Our research team at ICCB was composed of all authors and 2 data‐collection assistants. We carried out face‐to‐face interviews with attendees, during which the Q survey provided the main stimulus. Respondents were selected purposively, rather than following conventional inferential statistical sampling aims, in order to capture the widest possible range of views (Watts & Stenner 2012). Four aspects drove our recruitment: people with a range of seniority, from thought leaders to junior conservationists; people with a known and distinct position on the debate (e.g., those who presented a relevant conference paper or referred to the debate); people without a known position on the debate who revealed in an initial conversation that they had a position; and people of both genders and from different sectors (e.g., academic and practitioner) and geographic origins. The team met daily throughout the congress to discuss progress and develop strategies to target underrepresented groups or perspectives until we judged that a sufficiently wide range of viewpoints had been captured, which was when responses represented both the existing published positions and a range of other perspectives on the debate. We also ensured that our 4‐fold recruitment objectives were achieved. Thirty Q sorts were completed (Table 2). Respondents were informed that their responses would be anonymized and were asked to represent their own views rather than those of their organization. Permission to conduct the survey was obtained in advance from the organizers of ICCB. This research was subject to the ethical clearance procedure for research with human subjects at the University of Leeds.

**Table 2:** Composition of the sample of people interviewed about their views of the new‐conservation debate

Gender	Female	Male				
	12	18				
Continent	Europe	Africa	Asia	N America	Oceania	S America
	13	3	3	4	6	1
Sector	NGO	Academia				
	13	17				
Self‐identify as:	Researcher	practitioner	both			
	18	5	7			

### The Interviews

**Figure 1:**
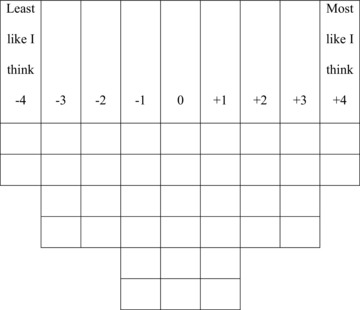
The Q methodology grid used in the study. Respondents were asked to allocate statements to cells reflecting their relative agreement with each statement.

All interviews were conducted in a quiet place away from other people. After an initial explanation of the project and the method, respondents completed the Q survey, sorting the statements onto the grid (Fig. 1). We emphasized that the method measures the extent to which respondents agree with each statement relative to all the other statements, rather than gauging an absolute level of agreement. The grid and our instructions covered the range from *most like I think* to *least like I think*, and we encouraged respondents first to gather statements into three piles. Two of these represented statements at the ends of the salience continuum, whereas the third was for statements of lower or intermediate salience. Respondents were then asked to distribute statements onto the grid from these piles. During the interview, respondents were encouraged to explain the rationale behind their sorting. This yielded complementary qualitative data recorded in writing by the researchers. Where respondents had questions about statements, the researcher gave limited help to explain the meaning of the statement while aiming not to bias the respondent.

Theory suggests that Q methodology grids should follow a normal distribution (Watts & Stenner 2012). Respondents were not constrained to follow the normal distribution shown on the grid but were encouraged to follow it as closely as possible. Rather than being a requirement of statistical analysis, this encourages respondents to prioritize statements, thereby revealing what is really salient to them (McKeown & Thomas 1998; Watts & Stenner 2012). Fifteen of the 30 respondents did not constrain their responses exactly to the normal distribution.

### Q Analysis

The Q sorts were analyzed using PQMethod software. A Q analysis involves three statistical procedures applied sequentially: correlation, factor analysis (here centroid analysis), and computation of factor scores (Watts & Stenner 2012). We rotated 3 factors following criteria in Watts and Stenner (2012). We based this decision on our judgment of the quantitative results of the analysis and our qualitative interpretation derived from our understanding of the respondents and their views. We used a varimax analysis and PQMethod's statistical threshold to automatically flag respondent Q sorts to factors. Five respondents were not flagged for any 1 factor. Following the quantitative stages, the analysis becomes more interpretive of the factors and is understood through representative Q sorts generated for each factor during the analysis (which represent the common ordering of statements for Q sorts associated with this factor) (Table 3). Table 3 was devised to help readers interpret differences between factors. We interpreted the factors themselves and the consensus statements, which did not distinguish between any pair of factors. We recognize interpretation in Q is somewhat subjective (Eden et al. 2005). Where we refer to qualitative interview data in the results section, it derives from a respondent belonging to the factor described.

**Table 3:** Numerical representations of factors and *z* scores and normalized *Q* scores (corresponding with the grid in Fig. 1) for each statement in the Q set.^a^

		Factor 1		Factor 2		Factor 3		
Statement number		norm	*Z*	norm	*Z*	norm	*Z*	Dist and cons^a^, ^b^
1	Humans are separate from nature not part of it.	−4	−1.88	−4	1.49	−4	−2.23	
2	Win‐win outcomes for people and nature are rarely possible.	−3	−1.06	−4	−1.63	0	0.02	F1, F2, F3
3	Conservation will only succeed if it provides benefits for people.	0	0.05	1	0.61	2	1.11	F2, F3
4	Conserving nature for nature's sake should be a goal of conservation.	0	0.33	3	1.17	−1	−0.30	F1, F2, F3
5	Conservation must benefit poor people because to do so is an ethical imperative.	1	0.69	1	0.41	0	0.20	cons
6	To achieve conservation goals, the environmental impact of the world's rich must be reduced.	4	1.43	2	0.82	1	0.49	F1
7	Conservation actions should primarily be informed by evidence from biological science.	−1	0.70	1	0.53	−1	−0.31	F2
8	It is acceptable for people to be displaced to make space for protected areas.	−1	−0.60	0	−0.03	−3	−1.73	F1, F2, F3
9	Pristine nature, untouched by human influences, does not exist.	3	1.20	−2	−1.13	3	1.38	F2
10	Strictly protected areas are required to achieve most conservation goals.	−2	−1.00	2	0.69	−4	−1.83	F1, F2, F3
11	There is a risk that highlighting human domination of the planet may be used to justify further environmental damage.	0	−0.45	−1	−0.57	−2	−0.42	cons
12	Nature often rebounds from even severe perturbations.	0	−0.13	−1	−0.30	1	0.48	F3
13	Conservation goals should be based on science.	0	−0.38	3	1.83	2	0.82	F1, F2, F3
14	Protecting nature for its own sake does not work.	−2	−1.04	−3	−1.38	1	0.22	
15	There is no significant conservation value in highly modified landscapes.	−1	−0.84	−3	−1.43	−3	−1.32	cons
16	Conservation will only be a durable success if it has broad public support.	1	0.72	3	1.39	2	1.07	
17	Conservation should work with, not against, capitalism.	−3	−1.16	−1	−0.36	1	0.29	F1, F2, F3
18	Working with corporations is not just pragmatic; they can be a positive force for conservation.	−1	−0.55	1	0.31	3	1.18	F1, F2, F3
19	To achieve conservation goals, human population growth must be reduced.	0	0.10	2	0.79	1	0.51	
20	Human affection for nature grows in line with income.	−3	−1.13	−3	−1.30	−2	−1.00	cons
21	Advancing the well‐being of all people should be a goal of conservation.	1	0.94	1	0.37	0	0.05	F1
22	Conservation should seek to reduce the emotional separation of people from nature.	3	1.14	−1	−0.54	0	0.12	F1, F2, F3
23	Conservation goals should be based on ethical values.	4	1.33	1	0.40	−1	−0.26	F1, F2, F3
24	Maintaining ecosystem processes should be a goal of conservation.	3	1.19	4	1.84	4	1.61	
25	Economic arguments for conservation are risky because they can lead to unintended negative conservation outcomes.	1	0.74	0	0.12	0	0.08	F1
26	Plural rationales for conservation weaken the conservation movement.	−4	−1.65	−1	−0.77	−3	−1.59	F2
27	Conservation messages promoting the benefits of nature to humans are less effective than those that emphasise the value of nature for nature's sake.	−1	−0.67	−2	−0.92	−2	−0.78	cons
28	There is a risk that economic rationales for conservation will displace other motivations for conservation.	2	0.98	0	0.14	−1	−0.17	F1
29	Conservation communications are more effective when they use doom and gloom rather than positive messages.	−2	−0.96	−3	−1.31	−3	−1.67	
30	Giving a voice to those affected by conservation actions improves conservation outcomes.	1	0.81	2	0.92	3	1.25	cons
31	To achieve its goals, conservation should seek to reform global trade.	2	1.10	−1	−0.37	1	0.36	F1, F2, F3
32	Non‐native species offer little conservation value.	−1	−0.71	−2	−0.95	−1	−0.35	cons
33	Human impact on nature grows in line with incomes.	1	0.89	0	0.15	−2	−0.48	F1, F2
34	Maintaining biological diversity should be a goal of conservation.	2	1.09	4	2.01	3	1.23	F2, F3
35	Conservation will only be a durable success if it has the support of corporations.	−3	−1.29	0	−0.28	0	−0.13	F1
36	Conservation should seek to do no harm to poor people.	2	1.13	0	0.27	4	1.57	F2
37	Giving a voice to those affected by conservation action is an ethical imperative.	3	1.28	3	1.01	2	0.77	cons
38	The best way for conservation to contribute to human well‐being is by promoting economic growth.	−2	−0.96	−2	1.01	−1	−0.26	F3

a Blank cells indicate statements that were neither consensus statements nor statistically significant in distinguishing between factors.

b “Cons” indicates consensus statements, otherwise indicates distinguishing (dist) at *p*< 0.05, and for which factor.

## Results

The Q statement numbers, normalized Q score for that statement for that factor, are in parentheses and distinguishing statements (ranked in a significantly different way in one or both other factors [Watts & Stenner 2012]) are marked with an asterisk.

### Factor 1

Factor 1 was associated with 9 respondents and was primarily distinguished by scepticism about markets, corporations, and capitalism; strong relative disagreement was displayed that conservation should work with capitalism (17^*^, −3). There was concern that economic rationales displace other motivations for conservation (28^*^, 2) and lead to unintended consequences (25^*^, 1). More generally, plural rationales were thought to strengthen conservation (26, −4). Corporations were not considered a positive force for conservation (18^*^, −1), and their support was not considered essential (35^*^, −3). As one respondent noted, corporations are “unlikely to fully support conservation objectives” (interview 9). There was relative disagreement that economic growth is the best way to promote human well‐being (38, −2) and reform of global trade was considered necessary (31^*^, 2).

This factor conveyed strong concern with the environmental impact of the world's rich (6^*^, 4) and less concern with overall population growth (19, 0) relative to factors 2 and 3. Associated respondents believed conservation should do no harm to poor people (36, 2) and should seek to improve the well‐being of all humans (21^*^, 1). These goals were higher priorities than conserving nature for nature's sake (4^*^, 0) but slightly lower than conserving ecosystem processes (24, 3) and biodiversity (34, 2). This factor conveyed ambivalence about whether conservation can be successful only by benefiting the poor (3^*^, 0). This factor consistently did not favor traditional wilderness‐focused conservation and conveyed the sense that pristine nature does not exist (9, 3) and that humans are not separate from nature (1, −4).

This factor promoted the idea that ethical values (23^*^, 4) are more important than science (13^*^, 0) in setting goals. Several respondents opined that the goals themselves are ethical statements. One noted that “science should inform how you do things in conservation, but not necessarily the goals” (interview 18). Biological evidence was not considered the most important source of evidence (7, −1). Unlike other factors, factor 1 was characterized by the idea that conservation should reduce human's emotional separation with nature (22^*^, 3).

### Factor 2

Factor 2 was associated with nine respondents. The most salient statements of factor 2 related to the importance of conserving biodiversity (34^*^, 4) and ecosystem processes (24, 4) as goals of conservation. The factor was distinctly biocentric, prioritizing nature for nature's sake (4^*^, 3) and rejecting the idea that protecting nature for its own sake does not work (14, −3). Human well‐being as a conservation goal was not a strong priority (21, 1), but this factor considered outcomes that mutually benefited nature and humans as often as possible (2^*^, −4). Together, these 2 elements and the placement of statement 3^*^ (1), regarding an instrumental rationale for conservation providing benefits to local people, characterized human well‐being as an important secondary objective of conservation. Factor 2 was pragmatic relative to an interest in plural rationales (26^*^, −1), and public support for conservation was regarded as a priority (16, 3). The use of doom and gloom messages was strongly rejected (29, −3).

The placement of statements 15 and 32 showed that value in nature was considered to be everywhere and that conservation should take place in all landscapes (e.g. “agricultural landscapes can have a very high conservation value” [interview 6]). However, some areas were considered pristine (9^*^, −2), a view that distinguished this factor. There was some interest in strictly protected areas (PAs) (10^*^, 2). This factor was strongly science‐oriented in terms of goal setting (13^*^, 3) and favored evidence from biological sciences (7^*^, 1).

Factor 2 conveyed a perceived need for reductions in population growth to achieve conservation goals (19, 2), for instance “I know it's controversial, but people are causing the problems and there are too many of them” (interview 5), and some concern about the environmental impacts of the rich (6, 2). In terms of how associated respondents considered local people and poverty, there was lower concern about doing no harm (36*, 0) and displacement of people by conservation action than in other factors (8^*^, 0). Although in the qualitative data respondents highlighted the need for appropriate consultation and consent from local communities (interview 15) and the need to avoid displacement, they also thought there may be cases where displacement could improve people's well‐being (interview 6).

Perspectives on economic arguments (25, 0; 28, 0), corporations (18^*^, 1), trade (31^*^, −1), and capitalism (17^*^, −1) were not priorities within this factor. This was coupled with the qualitative sense from one respondent that they did not have enough understanding of these issues to support strong views (interview 5). There was also pragmatism reflected in the idea that conservation needed to work with capitalism, but as one respondent stated: “that doesn't mean [capitalism] doesn't need to be changed” (interview 5).

### Factor 3

Factor 3 was associated with seven respondents and primarily distinguished by its relative optimism about corporations (18^*^, 3) and capitalism (17^*^, 1). Those aligned with this factor expressed relative disagreement that there is a risk of economic rationales displacing other motivations (28, −1) and neutrality about whether using economic arguments could lead to unintended consequences (25, 0). In the words of one respondent aligned with this factor, “Capitalism is not such a bad thing” (interview 29). Those aligned with this factor believed that reforming global trade is necessary (31^*^, 1) and that human population growth should be reduced (19, 1), but their views on these issues lay between the other factors’ positions. Respondents thought that impacts on nature do not grow in line with income (33^*^, −2).

Those aligned with this factor held strong views about the impact of conservation on people, believing it should do no harm to the poor (36, 4) and should not displace people to make way for PAs (8^*^, −3). The factor displayed more optimism than others about the contribution of economic growth to well‐being (38^*^, −1) and considered more strongly than others that conservation will only succeed if it benefits people (3^*^, 2). One respondent said when considering the well‐being statement (21), “No. The goal should be conservation” (interview 21). This factor displayed less optimism than others about the possibility of conservation mutually benefiting people and nature (2^*^, 0). One respondent said “I don't believe in this win‐win‐win, everyone wins. No. Some people will lose” (interview 29).

Those aligned with this factor believed pristine nature untouched by people does not exist (9, 3). Perhaps as a consequence, they expressed strong relative disagreement that strict PAs are required to achieve conservation goals (10^*^, −4). Biodiversity was slightly less of a priority for this factor than factor 2 (34, 3), and unlike the other factors, associated respondents did not see conserving nature for its own sake as a goal of conservation (4^*^, −1) of think this strategy works (14^*^, 1). The factor was positive about the role of science in goal setting (13^*^, 2) and saw the need for more than just biological science evidence in conservation (7, −1). Unlike factor 1, here ethical values were not seen as important for goal setting (23^*^, −1). As one respondent said, “maybe conservation has too many goals now” (interview 21).

Those aligned with this factor believed successful conservation requires broad public support (16, 2). They were fairly neutral on the need to reduce the emotional separation of people and nature (22^*^, 0). They also believed strongly that plural rationales do not weaken conservation (26, −3). One respondent said that “the inability to see others’ views, to see plurality of opinions and values is detrimental” (interview 23).

### Consensus Statements

There was relative consensus that significant value exists in highly modified landscapes (15), whereas non‐native species were generally thought to offer some conservation value (32). There was consensus in the weak relative disagreement with the idea that highlighting human domination of the planet may be used to justify further environmental damage (11). Consensus surrounded the idea that giving a voice to those affected by conservation actions improves conservation outcomes (30) and is an ethical imperative (37). There was consensus around a low salience ranking (+1 or 0) regarding whether conservation must benefit poor people as an ethical imperative (5) and relative disagreement with the proposition that human affection for nature grows in line with income (20). Relative consensus existed on the notion that conservation messages promoting anthropocentric rationales can be as effective as those emphasizing biocentric rationales (27). Finally, there was general agreement that maintaining biodiversity (34) and ecosystem processes (24) should be goals of conservation, but these did not meet the statistical criteria to be considered consensus statements.

## Discussion

This article provides the first published evidence of what a wider group of conservationists who have not actively participated in the public debate about the new conservation think about the issues raised and positions put forward within that debate. Our results suggest the existence of at least 3 distinct ways of thinking about these issues. Two of these positions were recognizably related to the traditional and new‐conservation positions described in the literature (factor 2 and factor 3, respectively), albeit with important distinctions. The third (factor 1) was strongly divergent from either of the positions described in the new‐conservation literature and included elements more closely resembling the positions on market‐based conservation found in the literature on neoliberal conservation. Below we offer descriptive labels for each factor. These are simplifications of the nuanced content of each factor, but they offer a useful shorthand to identify positions and facilitate further debate.

Factor 2 resembled the traditional conservation view most closely associated in this debate with the writing of Michael Soulé (2013; Miller et al. 2014), although with some important differences. As a result, we labeled it traditional conservation 2.0. Areas of overlap included a primarily biocentric motivation for conservation, a focus on conserving biodiversity and ecosystem processes, and a belief in the existence of pristine areas and in the value of biocentric arguments when communicating conservation. This factor placed a low priority on market‐based mechanisms and economic arguments for conservation, which resembles arguments put forward opposing the new conservation (e.g., McCauley 2015). However, factor 2 diverged from the standard traditional conservation position described in the literature. In particular (and in line with factors 1 and 3), it promotes the conservation of biodiversity wherever it is found, including of non‐native species and in highly modified landscapes, in contrast to the traditional conservation position that focuses strongly on pristine nature in strict PAs. This raises the question of whether the traditionalist position of authors such as Soulé (2013) and Wilson (2016) has relevance for many contemporary conservationists or represents an ultraorthodox view held by a small minority.

Factor 3 resembled the new‐conservation position most closely associated with the writing of Peter Kareiva and Michelle Marvier (Kareiva et al. 2012; Kareiva & Marvier 2012), although again there were important differences. As such, we labeled it nearly new conservation. Areas of overlap included a generally optimistic view of market‐based instruments in conservation, an interest in novel ecosystems, modified landscapes, and more pristine areas and a belief that science should play a strong role in conservation. Two areas of apparent distinction emerged between factor 3 and the standard new‐conservation positions. First, new‐conservation literature tends to adopt a primarily anthropocentric rationale for conservation in which benefiting people is an important goal in itself, whereas factor 3 was more concerned about avoiding harm to people than actually increasing their well‐being. This suggests factor 3 represented a more instrumental view of the importance of benefiting people as a means to an end rather than an end in itself. Second, factor 3 was fairly neutral on the importance of addressing a separation of people from nature, whereas Kareiva (2008: 2758), a key architect of the new conservation earlier argued that this separation “may well be the world's greatest environmental threat.”

Although factors 2 and 3 mapped fairly neatly onto positions described in the existing new‐conservation literature, factor 1 did not. It shared aspects of factor 3, including concern for biodiversity in modified and pristine landscapes and need to avoid harm to people. However, it strongly diverged from factor 3 on the role of corporations and market‐based instruments in conservation; it was critical of both. As such, we labeled it market scepticism. The position described by this factor is perhaps most closely aligned with those contained within critical social science scholarship on so‐called neoliberal conservation (e.g., Igoe & Brockington 2007; Brockington & Duffy 2010). There was also strong overlap with the position of Spash (2015) put forward in a recent article and presentation to the ICCB and with the social‐instrumentalism position described by Matulis and Moyer (2016). These critical arguments are almost absent from the literature that explicitly refers to the new‐conservation debate, despite appearing in mainstream conservation publications (e.g., Redford & Adams 2009) and being commonplace in the literature and conferences of the conservation social science community, which has academic audiences in geography, anthropology, political science, and other disciplines.

Our results have 2 important implications for the new‐conservation debate and broader thinking on future directions for conservation. First, there are more than two perspectives on what conservation is, why it matters, and how to do it. Others have pointed out that the new‐conservation literature creates a false dichotomy (Tallis & Lubchenco 2014), and our results support this. Critics argue that the debate has been dominated by established and influential figures from a narrow demographic, rather than representing the broader demographic of conservation researchers and practitioners (Tallis & Lubchenco 2014), and has been conducted in an overly adversarial manner (Marris 2014). Our qualitative data support this claim and the dissatisfaction with the tone and nature of the debate. One respondent working for an international NGO stated, “the modus operandi of the loudest voices [in the new‐conservation debate] is to provoke… It is a distraction from the real challenges the sector faces” (interview 23). Given that not all voices in conservation are present at the ICCB, particularly those of groups that have been historically marginalized in conservation debates, the range of opinions is undoubtedly even broader than what we captured.

Second, it is striking that we found a position (factor 1) that is almost completely absent from the new‐conservation literature. Nine of our respondents were associated with this perspective and a similar position was presented by Clive Spash, who received a standing ovation from large sections of the audience in a plenary debate at ICCB. This finding suggests there is a latent critical viewpoint on neoliberal conservation that is held by a large number of conservationists but not represented by the actions of most conservation organizations or the writing of scholars like Soulé, Kareiva, and Marvier. Previous Q‐method studies show similar resistance among some conservationists to market‐based conservation (Sandbrook et al. 2013a; Blanchard et al. 2016). Articles in mainstream conservation journals have critiqued the underlying premises of market‐based conservation (Redford & Adams 2009; Spash 2015), often authored by critical conservation social scientists. If such views are widespread, then there may be a ready audience for critical conservation social science scholarship among the conservation community, adding further weight to previous calls to improve the communication of ideas between these groups (Sandbrook et al. 2013b). To discover the prevalence of the viewpoints we identified, further research could build on this study by using survey methods designed to produce inferential results, focusing in particular on the conservation practitioner and non‐Anglophone communities that were less represented at the ICCB.

Conservation is many things to many people, and it is not surprising people do not agree about everything. Although divisions over the new conservation could be treated as an ecumenical matter (Marvier 2014), with different approaches more suitable in different contexts (Pearson 2016), there will be places where they will collide, and there will be important disagreements that are worth acknowledging and discussing (Sandbrook 2015). Matulis and Moyer (2016) argue that such “agonistic pluralism” is preferable to the “inclusive conservation” that others have called for (e.g. Tallis & Lubchenco 2014), which can stifle minority viewpoints. That said, we identified some important areas of consensus and shared ground among our respondents, such as a recognition of the value of modified habitats, the importance of conserving ecosystem processes, and the need to give a voice to local people. In what has often been an adversarial public debate, the existence of these points of agreement could provide platforms for constructive debate in the conservation community about areas of disagreement. Our findings provide a fuller and more nuanced understanding of the variety of views that exist. We hope this will improve the quality and tone of debates surrounding the future of conservation.

## Supporting information

A list of sources used to develop statements for Q Sort (Appendix S1) are available online. The authors are solely responsible for the content and functionality of these materials. Queries (other than absence of the material) should be directed to the corresponding author.
