# Supplementary material for: Understanding conservationists’ perspectives on the new‐conservation debate
Source: Conserv Biol. 2016 Nov 30;31(2):353–63. doi: 10.1111/cobi.12811 (PMC6849763; doi:10.1111/cobi.12811)
Supplement: Supplementary file 1 — A list of sources used to develop statements for Q Sort (Appendix S1) are available online. The authors are solely responsible for the content and functionality of these materials. Queries (other than absence of the material) should be directed to the corresponding author. [file COBI-31-353-s001.docx]

Appendix S1: List of sources used to develop statements for Q Sort. This search was undertaken in March 2015. The literature reviewed therefore does not include any papers published after this date.

Cafaro, P. 2014. A moral imperative to preserve other species: reply to Kareiva and Marvier. Biological Conservation:283.

Doak, D. F., V. J. Bakker, B. E. Goldstein, and B. Hale. 2014. Moving forward with effective goals and methods for conservation: a reply to Marvier and Kareiva. Trends in Ecology & Evolution **29**:132-133.

Doak, D. F., V. J. Bakker, B. E. Goldstein, and B. Hale. 2014. What is the future of conservation? Trends in Ecology & Evolution **29**:77-81.

Greenwald, N., D. A. Dellasala, and J. W. Terborgh. 2013. Nothing new in Kareiva and Marvier. BioScience **63**:241-241.

Hunter, M. L., K. H. Redford, and D. B. Lindenmayer. 2014. The Complementary Niches of Anthropocentric and Biocentric Conservationists. Conservation Biology **28**:641-645.

Kareiva, P. 2014. New conservation: setting the record straight and finding common ground. Conservation Biology **28**:634-636.

Kareiva, P., C. Groves, and M. Marvier. 2014. REVIEW: The evolving linkage between conservation science and practice at The Nature Conservancy. Journal of Applied Ecology **51**:1137-1147.

Kareiva, P., R. Lalasz, and M. Marvier. 2011. Conservation in the Anthropocene: beyond solitude and fragility. Love Your Monsters: postenvironmentalism and the anthropocene. Oakland: Breakthrough Institute.

Kareiva, P., and M. Marvier. 2012. What is conservation science? BioScience **62**:962-969.

Kirby, K. R. 2014. “New Conservation” as a Moral Imperative. Conservation Biology **28**:639-640.

Levin, P. S. 2014. New conservation for the anthropocene ocean. Conservation Letters **7**:339-340.

Marris, E. 2014. ‘New conservation’is an expansion of approaches, not an ethical orientation. Animal Conservation **17**:516-517.

Marvier, M. 2014. A call for ecumenical conservation. Animal Conservation **17**:518-519.

Marvier, M. 2014. New conservation is true conservation. Conservation Biology **28**:1-3.

Marvier, M., and P. Kareiva. 2014. The evidence and values underlying ‘new conservation’. Trends in Ecology & Evolution **29**:131-132.

Marvier, M., and P. Kareiva. 2014. Extinction is a moral wrong but conservation is complicated. Biological Conservation:281-282.

Marvier, M., and H. Wong. 2012. Resurrecting the conservation movement. Journal of Environmental Studies and Sciences **2**:291-295.

McEuen, A. B. 2014. Embrace new conservation. Frontiers in Ecology and the Environment **12**:322-323.

Miller, B., M. E. Soulé, and J. Terborgh. 2014. ‘New conservation’or surrender to development? Animal Conservation **17**:509-515.

Noss, R., R. Nash, P. Paquet, and M. Soulé. 2013. Humanity's domination of nature is part of the problem: A response to Kareiva and Marvier. BioScience **63**:241-242.

Petriello, M. A., and K. E. Wallen. 2015. Integrative reflections on the new conservation science debate. Biodivers Conserv **24**:1549-1551.

Soulé, M. 2013. The “New Conservation”. Conservation Biology **27**:895-897.

Soulé, M. 2014. Also seeking common ground in conservation. Conservation Biology **28**:637-638.

Tallis, H., and J. Lubchenco. 2014. Working together: A call for inclusive conservation. Nature **515**:27-28.
